# Supplementary material for: The Involvement of Cell-Type-Specific Glycans in Hydra Temporary Adhesion Revealed by a Lectin Screen
Source: Biomimetics (Basel). 2022 Oct 15;7(4):166. doi: 10.3390/biomimetics7040166 (PMC9589958; doi:10.3390/biomimetics7040166)
Supplement: Supplementary file 1 [file biomimetics-07-00166-s001.zip › biomimetics-1898903-supplementary.pdf]

biomimetics

MDPI

Article–Supplementary Materials

Supplementary: The Involvement of Cell-Type-Specific Glycans in *Hydra* Temporary Adhesion Revealed by a Lectin Screen

Sofia Seabra [1,2], Theresa Zenleser [2], Alexandra L. Grosbusch [2], Bert Hobmayer [2] and Birgit Lengerer [2,*]

[1] Institute Superior Técnico, University of Lisboa, Av. Rovisco Pais, 1049-001 Lisboa, Portugal
[2] Institute of Zoology and Center of Molecular Biosciences Innsbruck, University of Innsbruck, Technikerstr. 25, 6020 Innsbruck, Austria
* Correspondence: birgit.lengerer@uibk.ac.at

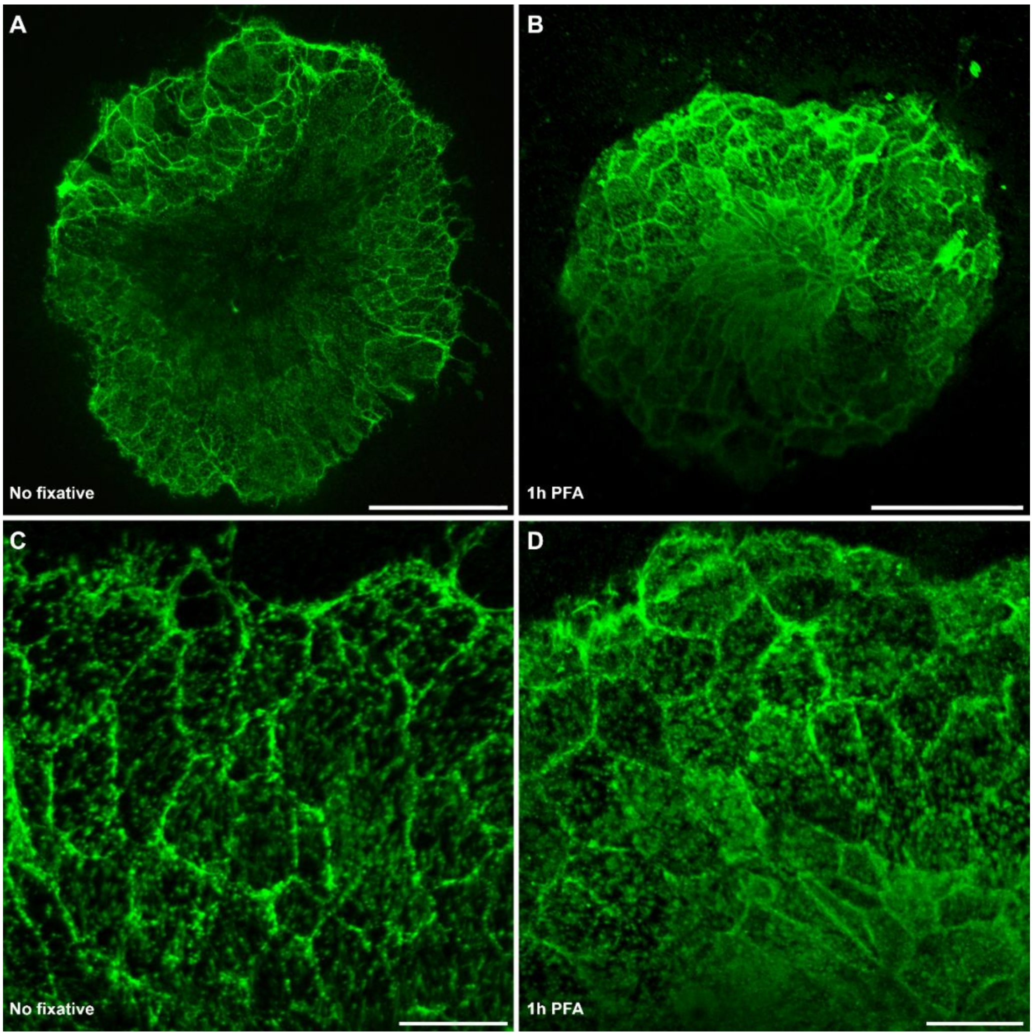

Figure S1: WGA labeling of *Hydra* footprints using (A,C) fresh (unfixed) footprints and (B,D) footprints fixed for 1 hour with PFA. (A,B) Footprint overview staining revealed the imprint of the cell borders, with a meshwork-like substructure. (C,D) Higher magnification revealed a similar substructure of the footprints for both fixation methods. The fixation method used is indicated in the images. Scale bars: (A,B) 100 µm; (C,D) 20 µm.

Table S1: Overview of lectin binding specificity according to the manufacturer Vector laboratories.

Lectin	Acronym	Preferred sugar specificity	Comments
**Phaseolus vulgaris erythro agglutinin**	PHA-E	Galβ4GlcNAcβ2Manα6(GlcNAcβ4)(GlcNAcβ4Manα3)Manβ4	
**Phaseolus vulgaris leuco agglutinin**	PHA-L	Galβ4GlcNAcβ6 (GlcNAcβ2Manα3)Manα3	
**Griffonia (Bandeiraea) simplicifolia lectin II**	GSL II	α or βGlcNAc	Is unique in its ability to recognize exclusively α- or β-linked GlcNAc residues on the nonreducing terminal of oligosaccharides.
**Lens culinaris agglutinin**	LCA	αMan, αGlc	Recognizes sequences containing αMan residues but recognizes additional sugars as part of the receptor structure, giving it a narrower specificity than Con A.
**Vicia villosa agglutinin**	VVL	GalNAc	Recognizes preferentially α- or β-linked terminal GalNAc, especially a single GalNAc residue linked to serine or threonine in a polypeptide.
**Erythrina crista-galli lectin**	ECL	Galβ4GlcNAc	Sialic acid substitution appears to prevent the lectin from binding.
**Pisum sativum agglutinin**	PSA	αMan, αGlc	Is nearly identical in structure and carbohydrate specificity to LCA.
**Griffonia (Bandeiraea) simplicifolia lectin I**	GSL I	αGal, αGalNAc	GSL I is a mixture of the five isolectins recognizing αGal and αGalNAc.
**Dolichos bilforus agglutinin**	DBA	αGalNAc	
**Wheat germ agglutinin**	WGA	GlcNAc	Preferable binds to dimer and trimers of GlcNAc. Can also bind terminal GlcNAc and chitobiose.
**Succinylated wheat germ agglutinin**	sWGA	GlcNAc	This derivative does not bind to sialic acid residues, unlike the native form.
**Datura Stramonium lectin**	DSL	(GlcNAc)2-4	Preferable binds to chitobiose or chitotriose over single GlcNAc residues.
**Lycopersicon esculentum (tomato) lectin**	LEL	(GlcNAc)2-4	
**Soybean agglutinin**	SBA	α>βGalNAc	Binds to oligosaccharide structures with terminal α- or β-linked N-acetylgalactosamine, and to a lesser extent, galactose residues.
**Ricinus communis agglutinin**	RCA I	Gal, GalNAc	Binds to Gal or GalNAc residues of membrane glycoconjugates.
**Ulex europaeus agglutinin 1**	UEA 1	L-Fuc	Binds to many glycoproteins and glycolipids containing α-linked fucose residues.
**Concanavaline A**	Con A	αMan, αGlc	Recognizes α-linked mannose present as part of a core oligosaccharide in many serum and membrane glycoproteins

**Elderberry bark Lectin**	EBL	Neu5Acα6Gal/ GalNAc	Binds preferentially to sialic acid attached to terminal Gal in α-2,6 and to a lesser degree, α-2,3 linkage. This lectin does not appear to bind sialic acid linked to GalNAc.
**Jacalin**	Jacalin	Galβ3GalNAc	Binds also in the presence of conjugated sialic acid.
**Maackia amurensis lectin II**	MAL II	Neu5Acα3Galβ4GalNAc	Binds only particular carbohydrate structures that contain sialic acid. Unlike SNA which seems to prefer structures with (α-2,6) linked sialic acid, MAL II appears to bind sialic acid in an (α-2,3) linkage.
**Peanut agglutinin**	PNA	Galβ3GalNAc	Does not bind in the precence of conjugated sialic acid.
**Solanum tuberosum (potatoe) lectin**	STL	(GlcNAc)2-4	Binds oligomers of GlcNAc and some bacterial cell wall oligosaccharides containing GlcNAc and N-acetylmuramic acid.
**Sophora Japonica agglutinin**	SJA	βGalNAc	

Sugar Abbreviations:

Fuc L-Fucose
Gal D-Galactose
GalNAc N-Acetylgalactosamine
Glc D-Glucose
GlcNAc N-Acetylglucosamine
Man Mannose
Neu5Ac N-Acetylneuraminic acid (sialic acid)

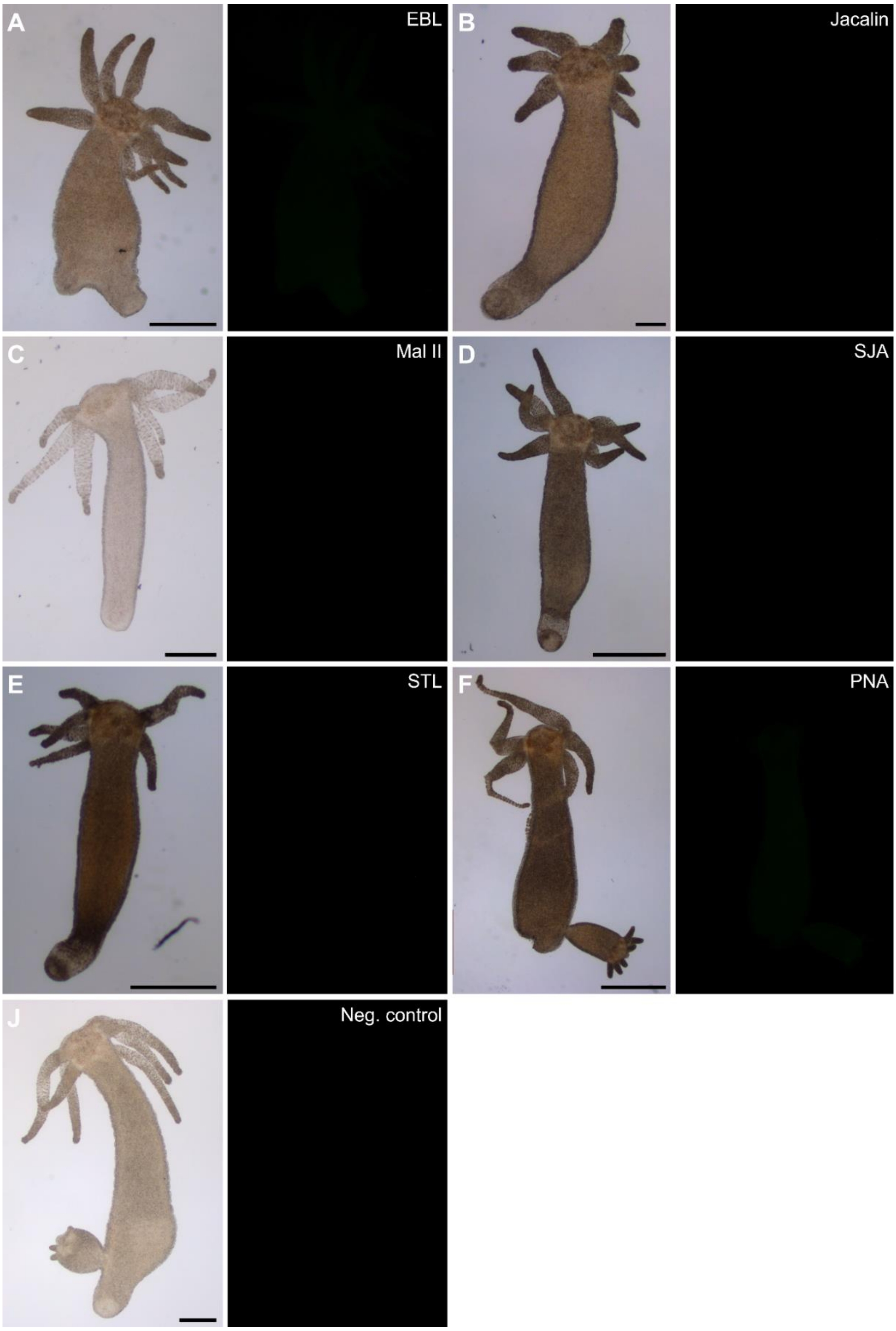

Figure S2: Lectin labeling of *Hydra* whole-mounts with (A) EBL, (B) Jacalin, (C) Mal II, (D) SJA, (E) STL, (F) PNA and (J) omitting a lectin and using only the Streptavidin-Dylight488 conjugate (negative control). Note that none of these lectins or the Streptavidin reacted with any *Hydra* tissue. The images show animals fixed overnight in PFA, but also Lavdowsky fixation led to the same results. Scale bars: (C,D,F,J) 500 µm; (A,B,E) 100 µm.

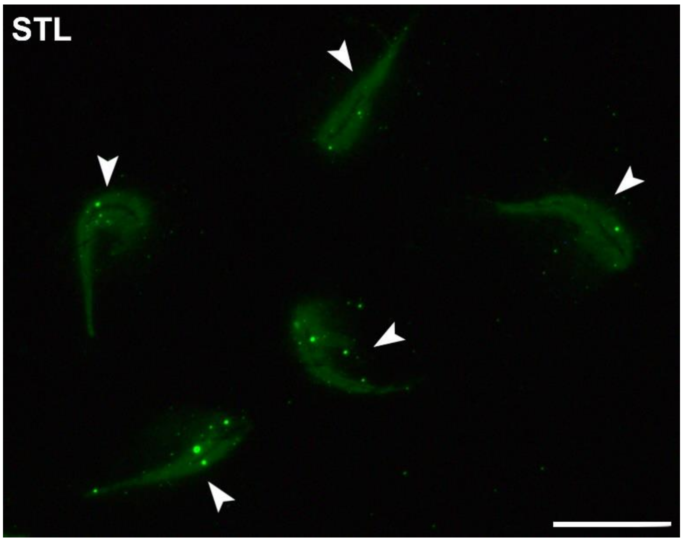

Figure S3: STL labeling of *Hydra* erupted nematocytes' tubules. Note that the staining is blurry, suggesting that the content of the capsules might contain glycans. Fixation with PFA for 1 hour at RT. Scale bar: 50 μm.

Whole mount labeling of lectins detecting the basal disc but not the footprints

Erythrina cristagalli lectin (ECL) labeled dot-like structures in the ectoderm similar to VVL (Figure S4A). In contrast to VVL, these structures were also present densely in the basal disc (Figure S4B). Additionally, after Lavdowsky fixation, nests of developing nematoblasts in the gastric region were weakly stained (Figure S4C). *Pisum sativum* agglutinin (PSA), *Griffonia (Bandeiraea) simplicifolia* lectin I (GSL I), and *Dolichos bilforus* agglutinin (DBA) labeled the gastric region, the tentacles, and the basal disc surface (Figure S4D-F). All three lectins labeled similarly, but with various intensities, the overall ectoderm surface (Figure S4D), the basal disc surface (Figure S4E), and the developing nematoblasts in the gastric region (especially after the Lavdowsky fixation). The main difference was observed in the tentacles, where PSA stained the surface and erupted nematocytes' tubules (Figure S4F), while GSL I and DBA strongly stained the capsules and the operculum of the nematocytes.

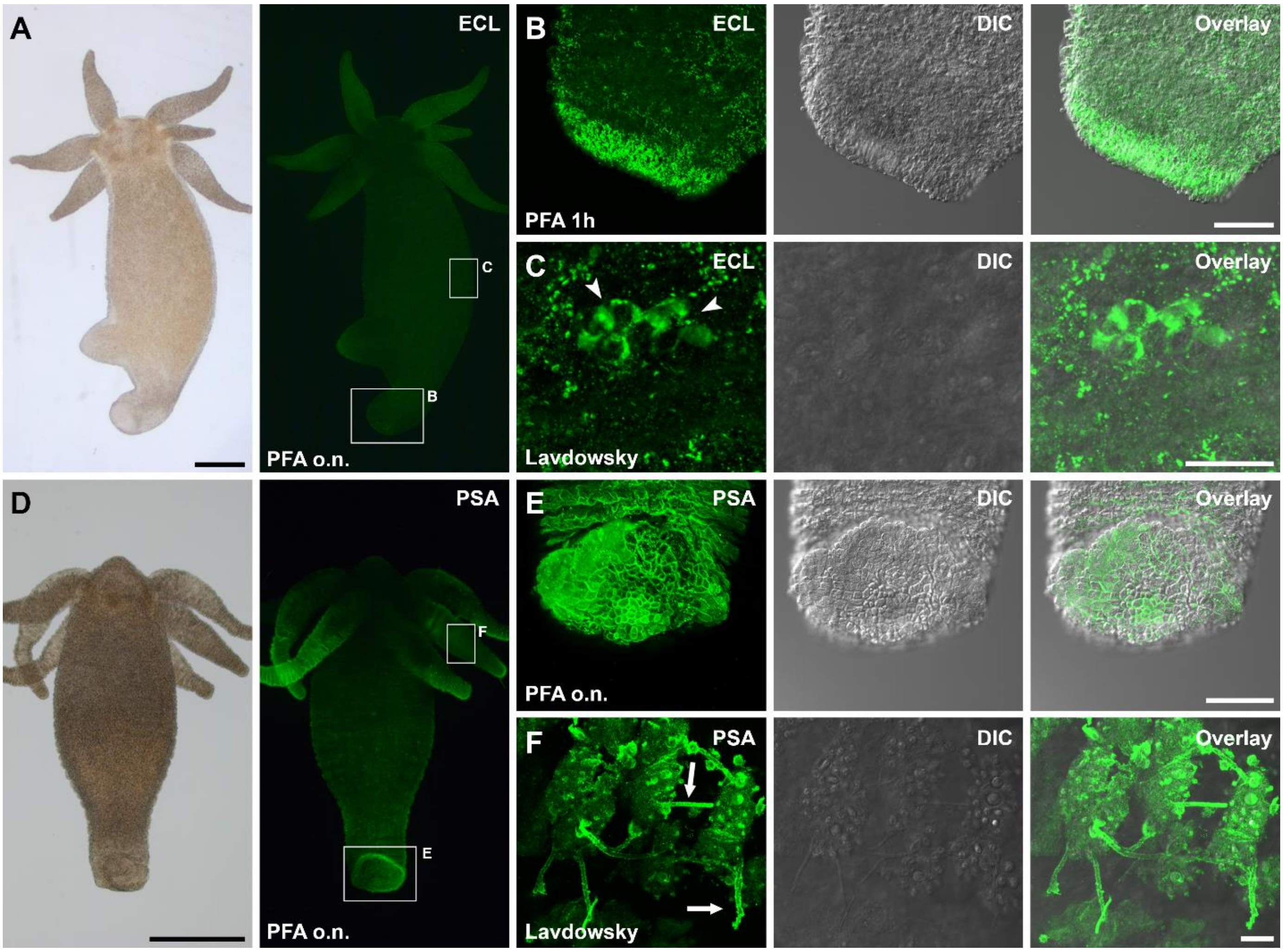

Figure S4: Lectin labeling of *Hydra* whole-mounts with (A-C) ECL and (D-F) PSA. (A-C) ECL labeling of a (A) whole-mount individual and (B) detailed view of the basal disc staining. (C) labeled nests of developing nematoblasts in the gastric region, highlighted with arrowheads. (D-F) PSA labeling of a (D) whole-mount individual and (E) detailed view of the basal disc staining. Labeled (F) surface and erupted nematocytes' tubules (indicated with arrows) in the tentacles. The fixation method used is indicated in the images. Scale bars: (A,D) 500 μm; (B,E) 100 μm; (C,F) 20 μm.

Whole mount labeling of lectins detecting the adhesive footprints

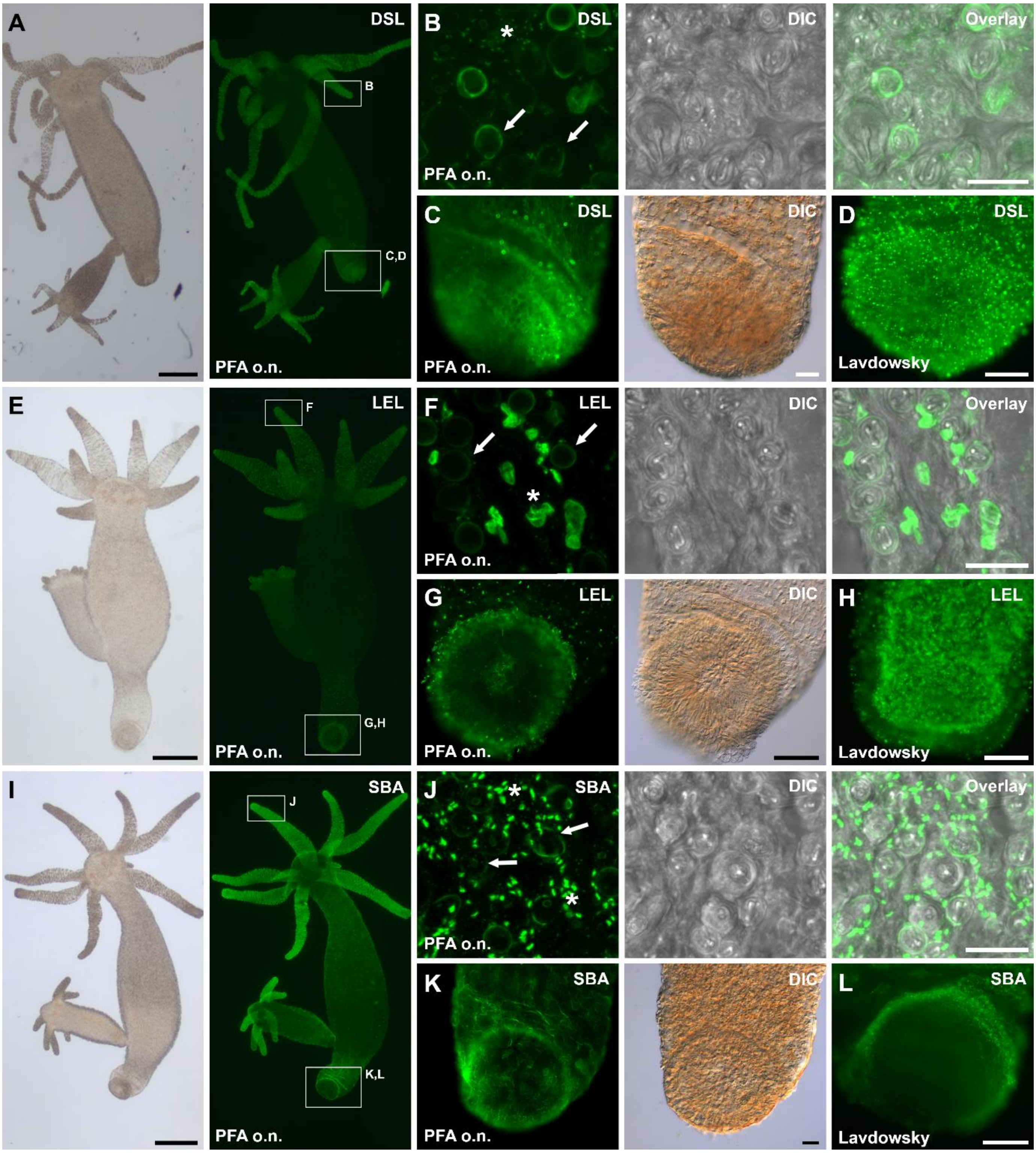

Figure S5: Lectin labeling of *Hydra* whole-mounts with (A-D) DSL, (E-H) LEL and (I-L) SBA. (A) DSL, (E) LEL and (I) SBA labeling of whole-mount individuals. (B,F,J) Ellipsoid structures in the surface and nematocyte capsules reacted in the tentacles, with all the presented lectins. (B,J) DSL and SBA additionally labeled the operculum. (C,D,G,H,K,L) No basal disc surface staining was detected for all presented lectins, except for LEL, using 1h PFA as fixative. The fixation method used is indicated in the images. Scale bars: (A,E,I) 500 µm; (C,D,G,H,K,L) 100 µm; (B,F,J) 10 µm.

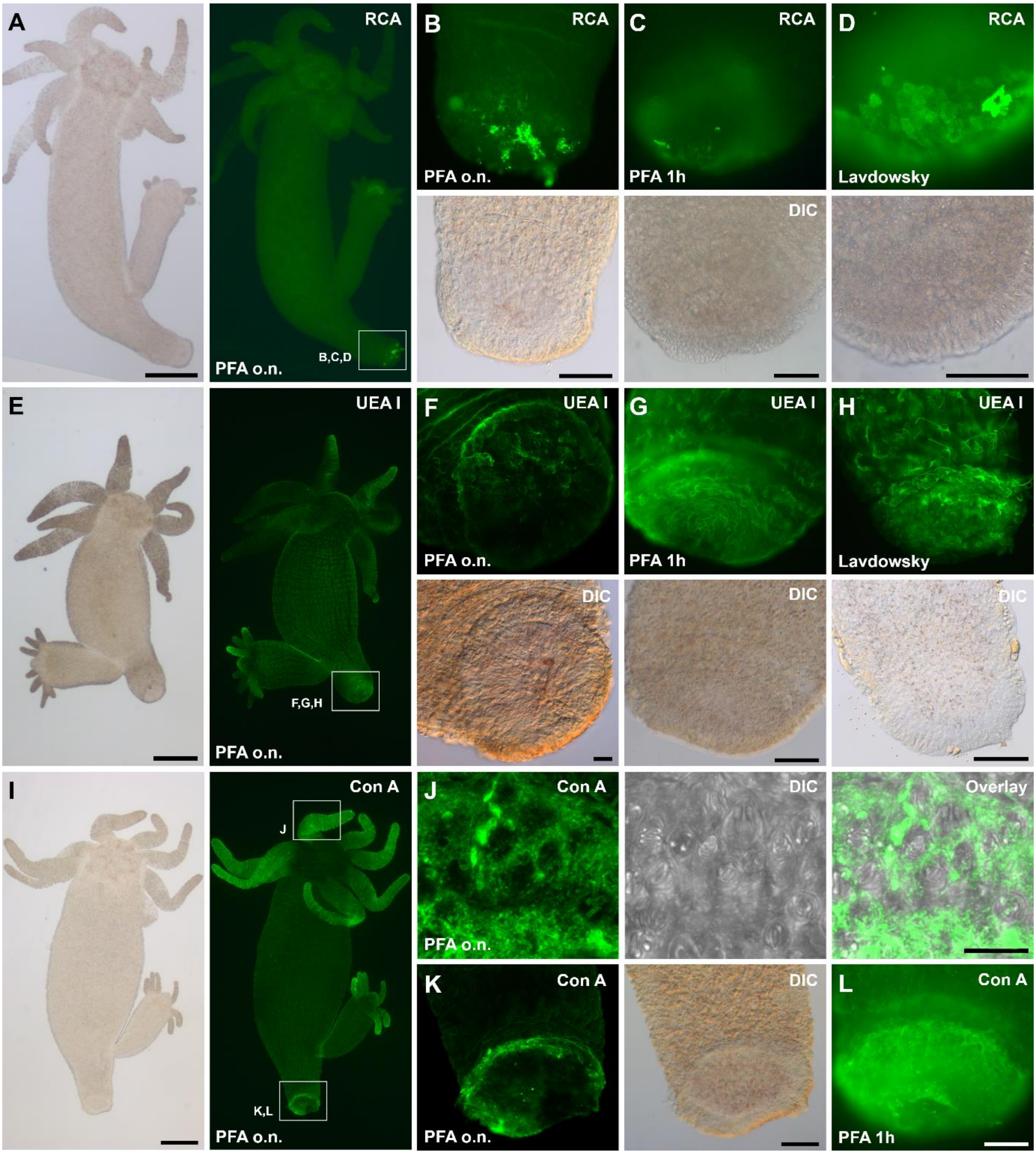

Figure S6: Lectin labeling of *Hydra* whole-mounts with (A-D) RCA, (E-H) UEA I and (I-L) Con A. (A) RCA and (E) UEA I labeling of whole-mount individuals, revealed overall ectoderm surface labeling. (B,C,D) Basal disc surface reacted with RCA, for all the different fixation methods. (F,G,H) Basal disc surface did not react with UEA I for any of the fixation methods. (I) Con A labeling of a whole-mount individual revealed weak reaction with the overall ectoderm surface. (J) Con A presented strong overall ectoderm surface staining, as did all structures of the nematocytes in the tentacles. (K,L) Basal disc surface intermediately labeled with Con A, for all fixation methods. The fixation method used is indicated in the images. Scale bars: (A,E,I) 500 µm; (B-D,F-H,K,L) 100 µm; (J) 10 µm.

Lectin labeling of universal and positional distinct Hydra cell types and associated structures

Three lectins labeled structures present throughout the whole animals (Figure S7). Phaseolus vulgaris erythro and leuco agglutinin (PHA-E and PHA-L), resulted in a similar labeling, reacting intermediately with the overall ectoderm surface of the animal (Figure S7A). Higher resolution revealed a strong reaction with the endoderm that appeared patchy throughout the gastric region. The labeled structures seemed to be associated with muscle fibers and followed the orientation of the endodermal radial actin filaments (Figure S7B). Additionally, in randomly distributed areas, the cell membranes of the ectoderm and endoderm were strongly stained (Figure S7C). This pattern occurred predominantly after the Lavdowsky fixation. With the Griffonia (Bandeiraea) simplicifolia lectin II (GSL II), the PFA and Lavdowsky fixations led to strikingly dissimilar results. Using PFA as fixative, intracellular structures, likely corresponding to vacuoles, were labeled throughout the whole animal body (Figure S7E,F). However, using Lavdowsky as a fixative, developing nematoblast nests in the gastric region and endodermal gland cells in the hypostome of the forming buds were stained (Figure S7G).

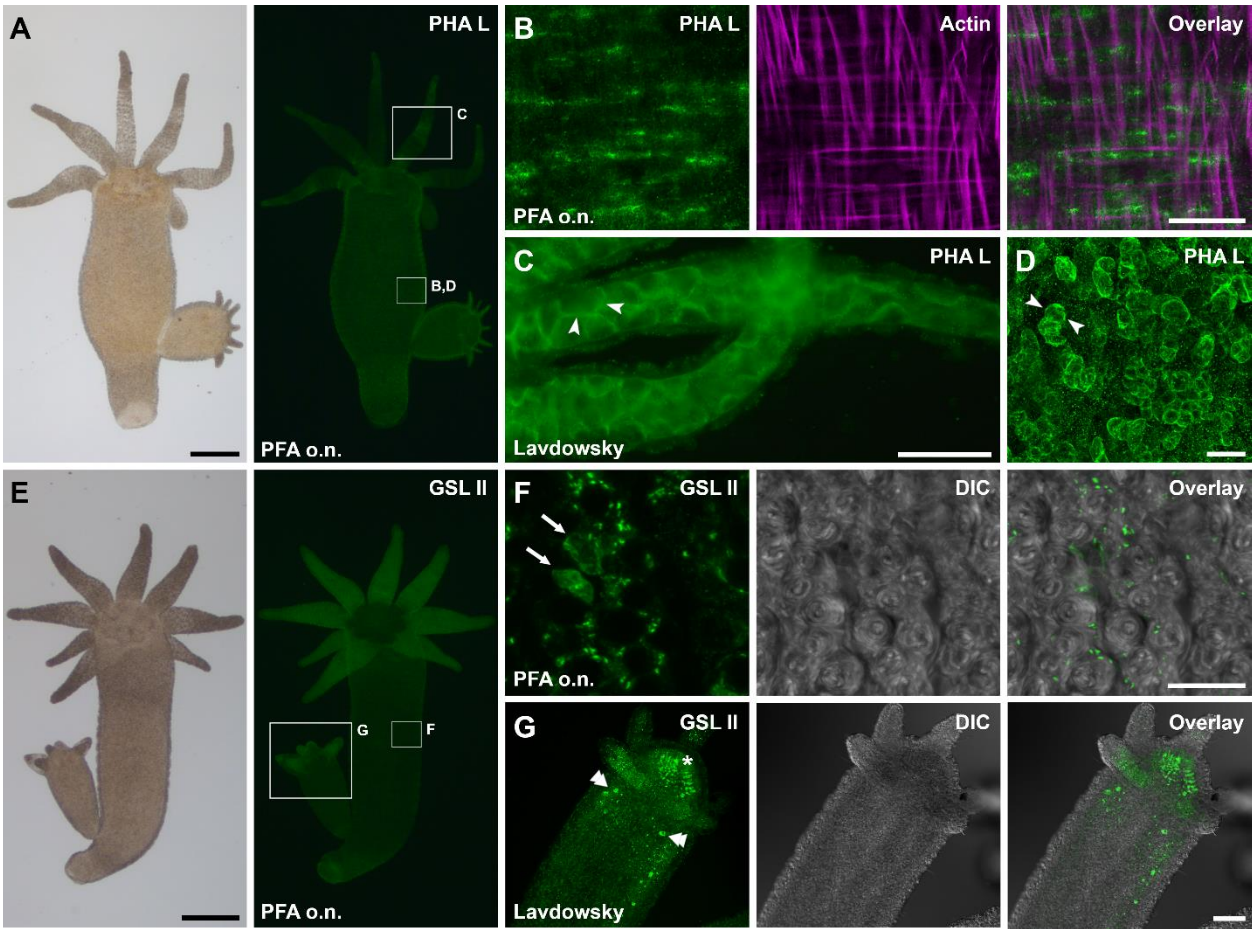

Figure S7: Lectin labeling of *Hydra* whole-mounts with (A-D) PHA-L and (E-G) GSL II. PHA-L labeling of a (A) whole-mount individual and (B) labeled structures in the gastric region, counterstained with phalloidin. Note that the PHA-L positive structures run parallel to the endodermal radial actin filaments. (C,D) Labeled cell membranes in the (C) tentacles and (D) gastric region, highlighted with arrowheads. (E-G) GSL II labeling of (E) a whole-mount individual and (F) detailed view of the intercellular structures in the tentacles. Arrows indicate the labeled intracellular structures. (G) Stained developing nematoblasts (indicated with double arrowheads) and endodermal gland cells (indicated with an asterisk) in the developing buds. The fixation method used is indicated in the images. Scale bars: (A,E) 500 μm; (B,D) 20 μm; (C) 50 μm; (F) 10 μm; (G) 100 μm.
